# Electronic and optical properties of silicene on GaAs(111) with hydrogen intercalation: a first-principles study

**DOI:** 10.1039/d1ra01959g

**Published:** 2021-04-30

**Authors:** Ting Yu, He Zhang, Dan Li, Yanwu Lu

**Affiliations:** Department of Physics, School of Science, Beijing Jiaotong University Beijing 100044 People's Republic of China ywlu@bjtu.edu.cn

## Abstract

In this paper, we investigated the electronic and optical properties of silicene on GaAs(111) substrates (silicene/HGaAs) on the basis of first-principles density functional theory. The hydrogen intercalation introduced substantially weakened the interaction between silicene and the GaAs(111) substrate and induced considerable bandgaps in silicene/HGaAs heterostructures. The effects of the interlayer spacing (*L*) between silicene and the substrate, silicene buckling height (*h*), biaxial strain (*ε*), and external electric field (*F*) on the electronic properties were also considered. Our results showed that the electronic properties of silicene/HGaAs heterostructures could be controlled by adjusting *L* and *h* and applying *ε* and an external *F*. Silicene/HGaAs heterostructures possessed the typical optical absorption properties of freestanding silicene and had high absorption coefficients. Besides, some strong peaks of absorption spectra and energy loss spectra existed in the ultraviolet light region, which showed that silicene/HGaAs heterostructures had evident enhancement in the ultraviolet light region. Results laid a theoretical foundation for the study of the electronic and optical properties and applications of silicene on semiconductor substrate devices.

## Abstract 2

In this paper, we investigated the electronic and optical properties of silicene on GaAs(111) substrates (silicene/HGaAs) on the basis of first-principles density functional theory.

## Graphical abstract

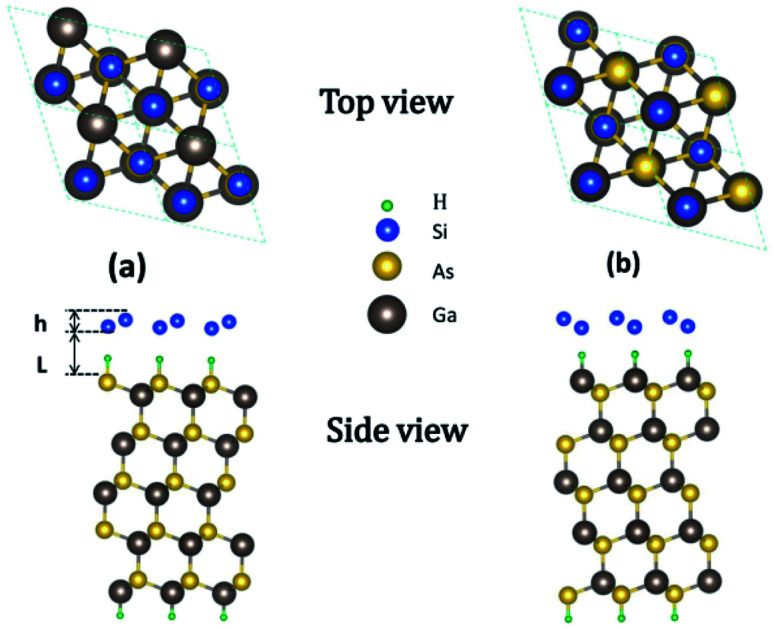

## 1. Introduction

Low-dimensional materials are important for the development of modern industry nowadays due to the demand for miniature electronic devices.^1–3^ Two-dimensional layered materials have high mechanical toughness, adjustable bandgap energy (*E*_g_) and optical transparency, and a high surface area ratio.^4^ These materials are ideal candidates for new nanoelectronic devices and high-sensitivity sensors. Silicene is a potential two-dimensional material among the low-dimensional materials that are currently popular in research. Silicene is a single-silicon monolayer with buckled honeycomb structure. The unique buckling structure of silicene brings several special physical properties, such as the quantum spin Hall effect,^5^ giant magnetoresistance,^6^ and strain-dependent heat conduction,^7^ which are difficult to achieve in pure graphene. The freestanding silicene layer exhibits a gapless semi-metallic characteristic with Dirac points, and its tunable *E*_g_ can be opened by an electric field, single-side surface adsorption of alkali, and interface interactions. In addition, given its high carrier mobility and easy integration into the modern Si-based device technologies, silicene is a promising material for high-speed electronic devices and has important application prospects in the field of microelectronic devices.

The buckled two-dimensional silicon is foreseen two decades ago (prior to graphene),^8^ and silicene was successfully grown on Ag(111) single crystals in 2012^9–11^ and the properties on this substrate were fully studied.^12–14^ The freestanding silicene layer is extremely unstable under normal temperature and pressure and is easily oxidized, thereby losing its structural characteristics when exposed to air.^15^ This surface sensitivity comes from its mixed sp^2^–sp^3^ characteristics.^16^ Its instability in the air has severely limited investigations of its experimental properties. A new encapsulated delamination transfer process conceived by Tao *et al.* in 2015 is expected to break the slow progress of the silicene device in the experiment.^17^ Tao *et al.* have encapsulated the silicene synthesized on the deposited Ag(111) sheet on a mica substrate with Al_2_O_3_ thin films and carried out the delamination transfer. The Ag substrate film must be preserved throughout the whole process. Otherwise, silicene degrades readily because of the exposed bottom surface. However, the Dirac cone of silicene is destroyed severely by the strong band hybridization between silicene and metallic substrates. This problem is expected to be solved using semiconductors as substrates for silicene. Semiconductor substrates can provide support for the silicene layer to ensure its stability and can weaken the interaction between interfaces. Therefore, identifying non-metallic substrates suitable to silicene is an important issue for realizing the devices based on silicene.^18^ In recent years, some studies about silicene and its analog germanene have turned from metal substrates into semiconductor substrates. Zhou *et al.* have reported growing graphene on SiC and induced *E*_g_ of 0.26 eV.^19^ Song *et al.* have grown graphene on a sapphire substrate and made a field-effect transistor.^20^ Chiappe *et al.* have recently successfully synthesized high flexural silicene on the surface of MoS_2_.^21^ These reports indicate that non-metal substrates have the potential for the experimental growth of silicene. Therefore, an in-depth study of the electronic and optical properties of silicene on semiconductor substrates is an important topic in the current field.

In this paper, GaAs(111) surfaces are selected as substrates for growing silicene, and hydrogen intercalation is used to weaken the interaction between silicene and GaAs(111) substrate. From the perspective of fundamentals and applications, this paper systematically investigates the stability and electronic and optical properties of silicene on the GaAs(111) substrate based on the first-principles density functional theory (DFT). The effects of interlayer spacing (*L*) between silicene and the substrate, buckling height (*h*), biaxial strain (*ε*), and external electric field (*F*) on its electronic properties are also considered. It provides theoretical supports for the growth of silicene on semiconductor substrates and the application of silicene devices in experiments.

## 2. Computational details and structural properties

All calculations were performed using the first-principles DFT as implemented in the Vienna *Ab initio* Simulation Package (VASP).^22^ Projector augmented waves^23^ were used in our calculations to describe the interaction between ion core and valence electrons, and the generalized gradient approximation (GGA) with the Perdew–Burke–Ernzerh (PBE) formula^24^ was used to approximate the energy related to electron exchange. In order to accurately describe the interface interaction of the heterostructures, the exchange–correlation function was performed using the van der Waals density functional (optB86b exchange–correlation form)^25^ to describe the interface interaction of the heterostructures accurately. A plane wave cutoff energy of 400 eV and Monkhorst–Pack (M–P) *k*-point meshes, *i.e.*, 11 × 11 × 1 for the structure optimization and 33 × 33 × 1 for the calculations of optical properties, are used. All geometric optimizations are performed, and the energy and the force convergence are 10^−5^ eV and 0.01 eV Å^−1^, respectively. Considering that the GGA under the DFT framework tends to underestimate bandgap *E*_g_, the hybrid functional HSE06 scheme^26^ is used to correct some calculations. A 1 × 1 unit cell slab with silicene placed on GaAs layers along the (111) direction is established, and the coverage rate of 50% hydrogen atoms is inserted to weaken the interaction between the silicene layer and the GaAs(111) surface. Five layers of GaAs atoms along the (111) direction are used to simulate the GaAs(111) surface, and the three bottom layers of GaAs atoms are fixed. The dangling bonds on the bottom of the GaAs(111) slab are saturated by pseudo-H atoms to recover bulk-like behaviors. A vacuum space of 15 Å is used to avoid the artifacts of the periodic boundary conditions.

GaAs with a direct *E*_g_ of 1.43 eV is a highly stable substrate for thin film growth and has a broad application potential. The choice of GaAs(111) substrate for silicene is motivated by many factors. (i) The lattice type of GaAs(111) is a hexagonal honeycomb structure matched with silicene. (ii) The lattice mismatch between silicene and GaAs(111) surface is only 3.5%. (iii) The silicene analog germanene can be epitaxially grown on the GaAs substrate.^27^ However, the silicene directly grown on the GaAs(111) substrate undergoes p- or n-type doping because of the charge transfer effect between the silicene and substrates.^28^ These states affect the electronic properties of silicene and cause the loss of the Dirac cone characteristic. Therefore, the hydrogen intercalation is introduced to weaken the interaction between silicene and the GaAs(111) substrate in this work. The mechanism of hydrogen insertion is similar to hydrogen corrosion and passivation. In experiments, Starke *et al.* have utilized hydrogen intercalation to separate graphene from the SiC(0001) surface successfully for the restoration of Dirac electronic properties.^29^ In 2013, Guo *et al.* have discovered that hydrogen intercalation can be used to separate the top Si atomic layer from Si(111) surface to form a freestanding silicene layer.^30,31^ In 2018, Prudkovskiy *et al.* have used the method of hydrogen intercalation to realize the processing technology successfully for nanostructure materials on a flat substrate in experiments.^32^ At present, this method is extensively studied and applicable to silicene/semiconductor systems.^33^ The hydrogen intercalation mechanism is expected to be an effective way to peel the silicene layer from substrates.

**Fig. 1:**
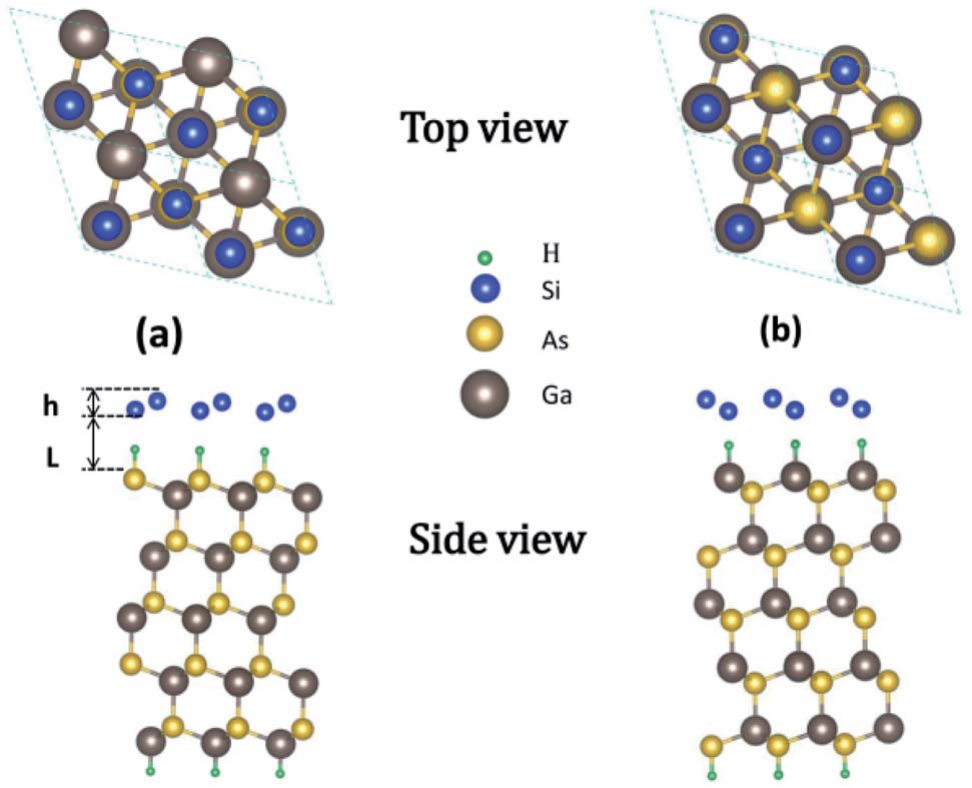
Top and side views of silicene/HAs–GaAs heterostructure (a) and silicene/HGa–GaAs heterostructure (b).

GaAs(111) has two different terminated surfaces, *i.e.*, Ga- and As-terminated surfaces.^34^ Given the different electronegativities of Ga and As atoms, their influences on the properties of silicene are also different. The configurations of silicene on HGaAs(111) have been discussed in our previous work including in ref. 28. The structures of silicene on As- and Ga-terminated GaAs(111) surfaces are shown in Fig. 1(a) and (b), respectively. In the initial structure, hydrogen atoms in the hydrogen intercalation are located directly below the downward-buckled Si atoms. After structural relaxation, H atoms remain on the surface of GaAs(111) and bonded with its surface atom (forming H–As– or H–Ga– intermediate surface), whereas silicene moves up perpendicular to the surface. The optimized structural parameters are listed in Table 1.

**Table 1:** Optimized structural parameters and binding-energy of silicene/HAs–GaAs and silicene/HGa–GaAs heterostructures. *L* is the distance between silicene and GaAs(111) surface, *L*_H_ is the distance between silicene and hydrogen intercalation, *h* is the buckling height of silicene, *a* is the lattice parameter and *E*_b_ is the binding energy

	*L* (Å)	*L*_H_ (Å)	*h* (Å)	*a* (Å)	*E*_b_ (eV/Si)
Silicene/HAs–GaAs	3.76	2.15	0.469	4.01	0.192
Silicene/HGa–GaAs	3.49	1.79	0.611	4.01	0.211

**Fig. 2:**
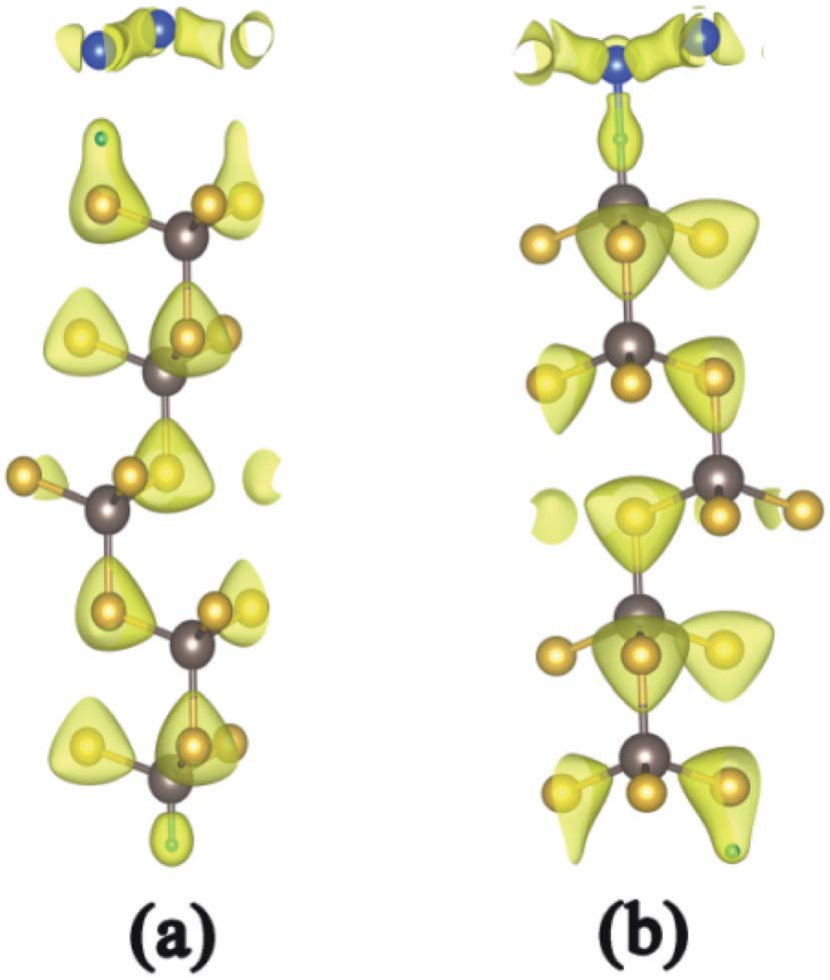
Charge density of silicene/HAs–GaAs heterostructure (a) and silicene/HGa–GaAs heterostructure (b).

The stability of the silicene/HGaAs heterostructures is assessed by defining the interface binding energy as:

1 *E*_b_ = (*E*_HGaAs_ + *E*_silicene_ − *E*_tot_)/*N*,

where *E*_HGaAs_, *E*_silicene_, and *E*_tot_ are the energies of the GaAs(111) substrate with hydrogen intercalation, isolated silicene, and silicene/HGaAs heterostructure, respectively, and *N* is the number of Si atoms in the silicene layer. The binding energy describes the energy obtained by placing an isolated silicene layer on the GaAs(111) substrate. The binding energies are 0.192 and 0.211 eV per Si atom (marked as eV/Si) for silicene on As- (silicene/HAs–GaAs) and Ga-terminated (silicene/HGa–GaAs) GaAs(111) surfaces, respectively (Table 1). By contrast, the silicene/HGa–GaAs heterostructure is more stable than the silicene/HAs–GaAs heterostructure. The binding energies of the two heterostructures are 3–4 times over the *E*_b_ (about 10^−2^ eV) under the action of van der Waals interactions. Fig. 2 shows the charge density of the two heterostructures. Fig. 2(a) presents that the Si atom in the silicene layer on the As-terminated surface does not form a covalent bond with the substrate surface (HAs–), which indicates that the strength of the interaction between the silicene layer and the substrate is between the van der Waals force and covalent bond. As for the silicene on the Ga-terminated surface, Fig. 2(b) shows the formation of a covalent bond between the Si atom and the substrate surface (HGa–). This difference is caused by the different electronegativities of As and Ga atoms (*i.e.*, 2.0 and 1.5, respectively). Given its high electronegativity, the As atom is strongly hybridized with the H atom orbital. Thus, the orbital hybridization effect of silicene and hydrogen intercalation is remarkably suppressed, thus forming an interaction between van der Waals forces and covalent bonds. By contrast, the H atom on the Ga-terminated surface is strongly hybridized with the Si atom orbital in the silicene layer, thus forming a covalent bond.

The *h* values of the silicene layer affect the interaction between silicene and the substrate (0.469 Å on As-terminated GaAs(111) surface, 0.611 Å on Ga-terminated GaAs(111) surface). The *h* of silicene on the As-terminated surface is close to that of the freestanding silicene layer (0.44 Å). In general, the large *h* indicates a strong interaction between silicene and the substrate, which increases the possibility of losing the Dirac cone and does not conduce to the silicene epitaxial growth in experiments.^18^

## 3. Results and discussion

### 3.1 Electronic properties

**Fig. 3:**
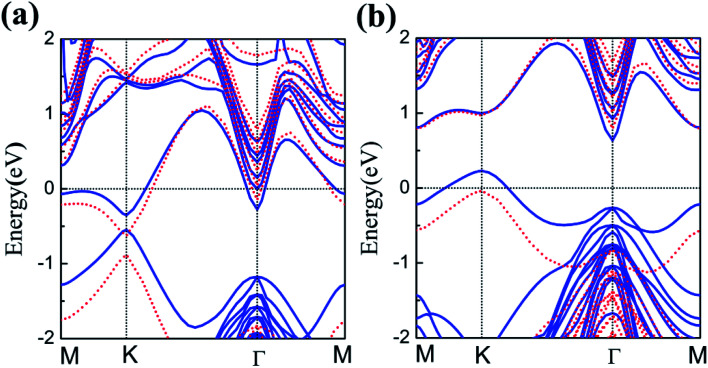
The band structures of silicene/HAs–GaAs heterostructure (a) and silicene/HGa–GaAs heterostructure (b). Blue solid lines are the results of GGA calculations, and red dashed lines are the results of HSE06 calculations.

The energy band structures of silicene on GaAs(111) surfaces along the Brillouin zone are shown in Fig. 3(a) and (b). The calculation results of the GGA exchange−correlation and the HSE hybrid functional are indicated by blue solid lines and red dashed lines, respectively. In Fig. 3(a), the silicene/HAs–GaAs heterostructure exhibits a direct bandgap at the high symmetry K point in the Brillouin zone. The *E*_g_ values for GGA calculation and HSE correction are 0.20 and 0.24 eV, respectively. The existence of the direct bandgap indicates that the interaction between the silicene layer and the As-terminated GaAs(111) surface is largely weakened by the hydrogen intercalation, resulting in no electronic orbital hybridization between Si atoms in the silicene layer and the substrate surface. The weak interaction maintains the Dirac characteristic of silicene and induces a considerable bandgap. For the silicene/HGa–GaAs heterostructure, indirect *E*_g_ values with valence band maximum (VBM) at the K point and the conduction band minimum (CBM) at the Γ point are observed (Fig. 3(b)). The *E*_g_ values for the GGA calculation and HSE correction are 0.41 and 0.82 eV, respectively. The hydrogen intercalation also induces the *E*_g_ of silicene on the Ga-terminated surface, but the dispersion characteristic of the Dirac cone is lost because of the covalent bond between Si and H atoms on the Ga-terminated surface. The downward buckling Si atoms form sp3 hybridization causing silicene to lose the Dirac electronic characteristics of sp^2^ hybridization. This result also indicates that the hydrogen intercalation has different effects on the electronic properties of silicene on different GaAs(111) terminated surfaces. Moreover, the Fermi energy level of silicene/HAs–GaAs heterostructure shifts upward into the conduction band, showing n-type semiconductor properties, whereas that of silicene/HGa–GaAs heterostructure shifts down into the valence band, showing p-type semiconductor properties. Evidently, this difference is also caused by the different electronegativities of Ga and As atoms.

The results of the HSE hybrid functional are compared with those of GGA calculations. HSE results correct the *E*_g_ by 0.04 and 0.41 eV for silicene/HAs–GaAs and silicene/HGa–GaAs heterostructures, respectively. Thus, GGA calculations are basically correct for the energy bands of silicene/HGaAs systems, especially for the silicene/HAs–GaAs heterostructure. The GGA calculations always underestimate the value of *E*_g_ but do not affect the qualitative analysis of the results. The calculations of GGA with PBE formula are adopted in the following discussion on the influence of the buckling height *h*, the interlayer spacing *L* between silicene and the substrate, biaxial strain *ε*, and external electric field *F* on electronic characteristics.

#### 3.1.1 Effects of buckling height and interlayer spacing

**Fig. 4:**
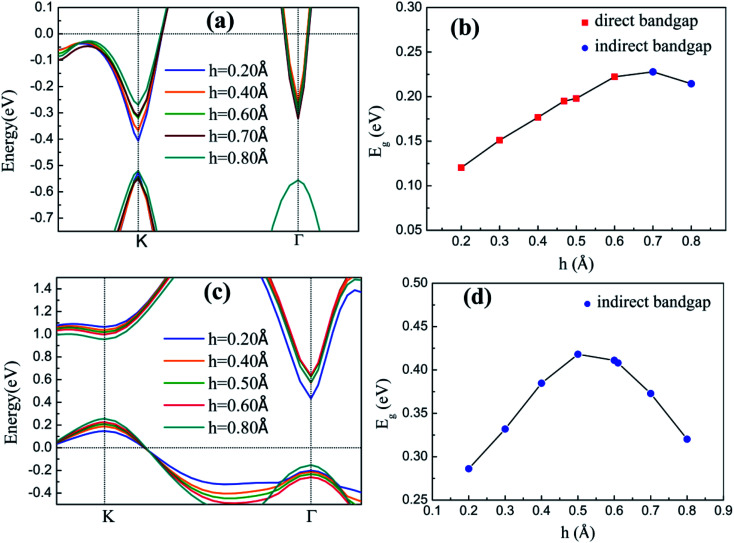
The band structures near Fermi level of silicene/HAs–GaAs heterostructure (a) and silicene/HGa–GaAs heterostructure (c), the bandgap *E*_g_ of silicene/HAs–GaAs heterostructure (b) and silicene/HGa–GaAs heterostructure (d) with different buckling height *h* of silicene.

The band structures of the two heterostructures at different buckling height *h* and interlayer spacing *L* values are calculated and discussed to understand the effects of *h* of silicene and *L* between silicene and substrate on the band characteristics. Fig. 4 shows the band structures and *E*_g_ values of two heterostructures at different *h* values. The variations in valence and conduction band edges of silicene/HAs–GaAs heterostructure are shown in Fig. 4 (a). Here, *L* between silicene and the As-terminated GaAs(111) surface is considered as the optimal relaxation value of 3.76 Å. The VBM is located at the K point and changes slightly with increasing *h*. The CBM is initially located at the K point and gradually moves upward with increasing *h*. When *h* increases to 0.70 Å, the CBM appears at the Γ point. In Fig. 4(b), the *E*_g_ of the silicene/HAs–GaAs heterostructure is initially a direct *E*_g_. As *h* increases, the *E*_g_ reaches the maximum of 0.23 eV at *h* of 0.7 Å, and transits from direct *E*_g_ to indirect *E*_g_. *E*_g_ decreases when *h* exceeds 0.7 Å. The VBM and CBM of the silicene/HGa–GaAs heterostructure varies with the *h* of silicene (Fig. 4(c)), and the *L* between silicene and Ga-terminated GaAs(111) surface is considered as the optimal relaxation value of 3.49 Å. The VBM of the heterostructure is located at K point and moves upward with increasing silicene *h*. The CBM is located at Γ point and gradually moves up with increasing *h*. When *h* is over 0.5 Å, the CBM begins to move down. Therefore, the *E*_g_ of silicene/HGa–GaAs heterostructure increases first and decreases with increasing *h*, as shown in Fig. 4(d). When silicene *h* approaches 0.5 Å, a maximum *E*_g_ of 0.42 eV, which is always an indirect *E*_g_, is observed.

**Fig. 5:**
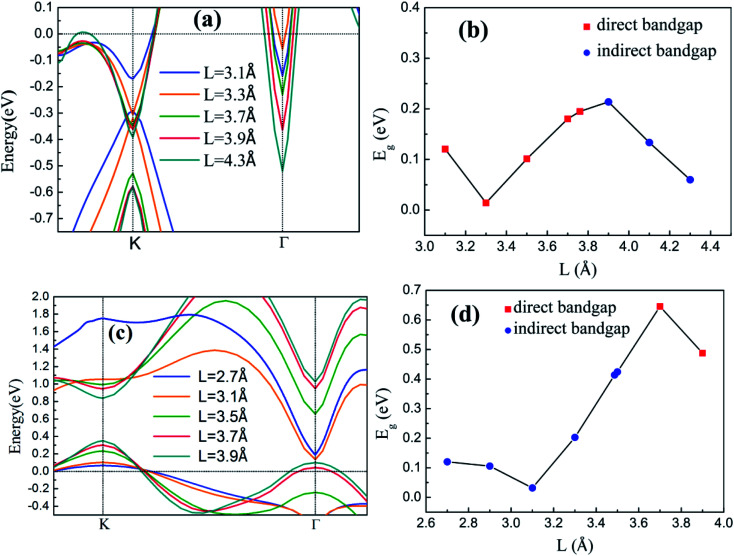
The band structures near Fermi level of silicene/HAs–GaAs heterostructure (a) and silicene/HGa–GaAs heterostructure (c), the bandgap *E*_g_ of silicene/HAs–GaAs heterostructure (b) and silicene/HGa–GaAs heterostructure (d) with different interlayer spacing *L* between silicene and GaAs(111) substrates.

The band structures and *E*_g_ of the silicene/HGaAs heterostructures as a function of *L* between silicene and the substrate are shown in Fig. 5. The variation in the valence and conduction band edges of the silicene/HAs–GaAs heterostructure with *L* is shown in Fig. 5(a). The silicene *h* is considered as the optimal relaxation value of 0.47 Å. The valence and conduction band edges move downward with increasing *L* at K and Γ points, respectively. A large *L* results in the evident upward shift of Fermi level. *E*_g_ presents an interesting variation with increasing *L* as shown in Fig. 5(b). *E*_g_ first decreases and then increases and reaches a maximum of 0.21 eV when *L* is 3.9 Å. When L is over 3.9 Å, *E*_g_ begins to decrease again and transits from direct *E*_g_ to indirect *E*_g_. The variation in the valence and conduction band edges of the silicene/HGa–GaAs heterostructure with *L* between silicene and the substrate are shown in Fig. 5(c), where the silicene *h* is considered as the optimal relaxation value of 0.61 Å. The VBM is located at K point and gradually moves up with increasing *L*. The CBM is located at Γ point, moves downward first, and then moves upward with increasing *L*. When L increases to 3.7 Å, the CBM moves to the K point. The variation in *E*_g_ is similar to that of the silicene/HAs–GaAs heterostructure. With increasing L, *E*_g_ decreases first and increases. The maximum *E*_g_ is 0.65 eV at *L* of 3.7 Å, and *E*_g_ is changed from indirect *E*_g_ into direct *E*_g_.

#### 3.1.2 Effect of biaxial strain

**Fig. 6:**
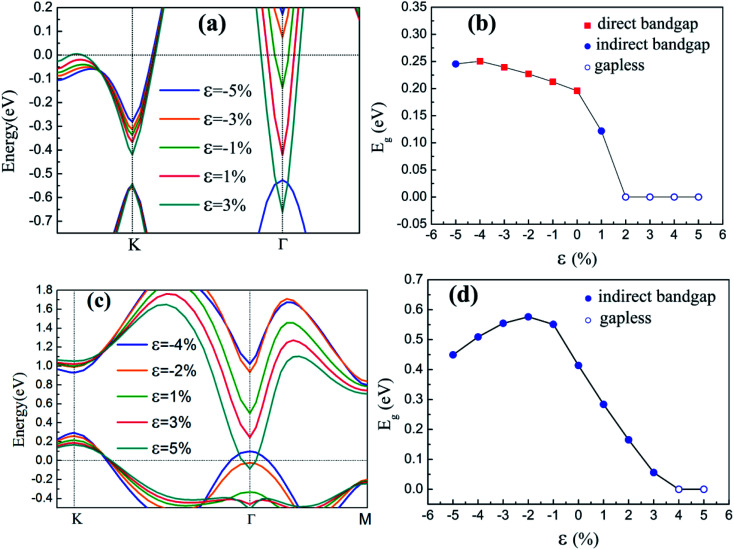
The band structures near Fermi level of silicene/HAs–GaAs heterostructure (a) and silicene/HGa–GaAs heterostructure (c), the bandgap *E*_g_ of silicene/HAs–GaAs heterostructure (b) and silicene/HGa–GaAs heterostructure (d) under different biaxial strain *ε*.

The effect of strain is crucial for the growth of silicene on any kind of substrate. The strain has become a powerful means to adjust the electronic structures and affect the carrier mobility in silicon-based materials^35,36^ and two-dimensional graphene materials.^37–39^ Considering the satisfactory match between silicene and GaAs(111) surfaces, a small biaxial strain *ε* ranging from −5% to 5% is applied, and band structures are obtained to evaluate the dependence of the electronic properties on *ε*. In this work, uniform stress is applied by changing the lattice parameters of the heterostructures. The biaxial strain is defined as:

2 *ε* = (*a*_1_ − *a*)/*a*,

where *a* and *a*_1_ are the lattice parameters of silicene/HGaAs heterostructures under the conditions of equilibrium status and external strain, respectively. Positive and negative values of *ε* represent tensile and compressive strains, respectively.^40^ The band structures around the Fermi level and *E*_g_ as a function of *ε* of the silicene/HGaAs heterostructures are shown in Fig. 6. It can be seen that under *ε*, the band dispersion of silicene/HAs–GaAs heterostructure around the K point almost remains intact, whereas the band dispersion around Γ point is remarkably affected in Fig. 6(a). The *E*_g_ under *ε* presents interesting characteristics. Under compressive *ε*, *E*_g_ first increases and then decreases with increasing *ε*. The maximum *E*_g_ is 0.25 eV at the *ε* of −4%, which is 25% larger than *E*_g_ without *ε*. When the compressive *ε* reaches −5%, the maximum *E*_g_ changes from a direct *E*_g_ to an indirect *E*_g_. Under tensile *ε*, the transition from direct to indirect *E*_g_ can also be observed at *ε* of 1%. When the tensile *ε* is over 1%, the CBM at Γ point moves down deeply, leading to the disappearance of the *E*_g_, and the silicene/HAs–GaAs heterostructure begins to exhibit the nature of metal. Fig. 6(c) shows that the VBM of the silicene/HGa–GaAs heterostructure is located at K point and that the CBM is located at Γ or M point. Compared with the band structure at K point, the band structure around Γ point is more sensitive to the *ε* change. *E*_g_ first increases and then decreases with increasing compressive *ε* (Fig. 6(d)). At *ε* of −2%, the maximum *E*_g_ is 0.58 eV, which is 41% larger than that without *ε*. Under tensile *ε*, *E*_g_ decreases with increasing *ε*. When the *ε* increases to 4%, *E*_g_ disappears, and the heterostructure transforms into a metal.

The inherent spin–orbit coupling in freestanding silicene induces a low *E*_g_ of 1.55 meV,^41^ and a little strain decreases *E*_g_.^42^ By contrast, the silicene on GaAs(111) surfaces can open large *E*_g_ values (0.25 eV for silicene/HAs–GaAs and 0.58 eV for silicene/HGa–GaAs) under *ε*. However, the two heterostructures can only withstand a little *ε*, and a large *ε* leads to loss of *E*_g_ and transformation into metals.

#### 3.1.3 Effect of external electric field

**Fig. 7:**
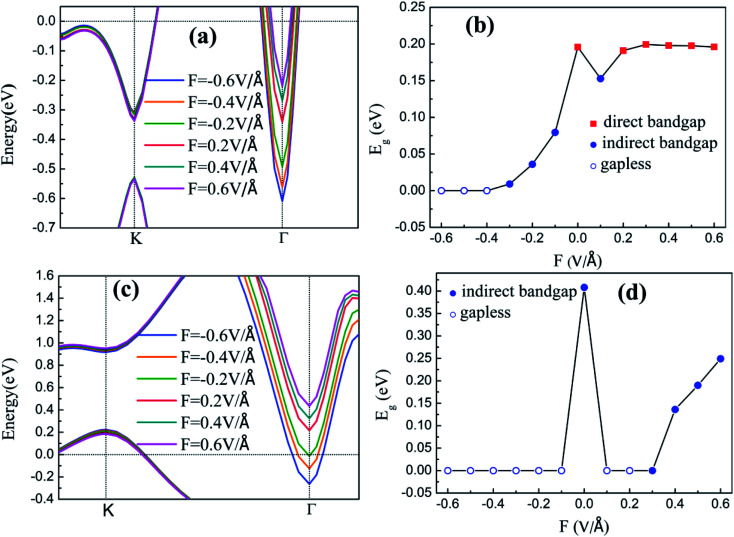
The band structures near Fermi level of silicene/HAs–GaAs heterostructure (a) and silicene/HGa–GaAs heterostructure (c), the bandgap *E*_g_ of silicene/HAs–GaAs heterostructure (b) and silicene/HGa–GaAs heterostructure (d) under different external electric fields *F*.

When heterostructures are used in nanodevice applications, they are often subjected to external electric fields *F*. Thus, the situation under the vertical *F* is discussed. The variation in the dispersion characteristics and *E*_g_ values of the two heterostructures under the *F* are shown in Fig. 7. Positive and negative vertical *F* are applied with an intensity of less than 0.6 V Å^−1^. As shown in Fig. 7(a) and (b), the VBM of the silicene/Has–GaAs heterostructure is located at the K point, and the CBM is located at K or Γ point, and the conduction band edges around Γ point are remarkably affected by *F*. When a negative *F* is applied, an indirect *E*_g_ exists in the silicene/HAs–GaAs heterostructure and decreases sharply with increasing *F*. When the *F* is −0.4 V Å^−1^, *E*_g_ disappears, resulting in metallic properties. When a positive *F* is applied, *E*_g_ first decreases, increases, and tends to saturate with increasing *F*. In addition, the transition between direct and indirect *E*_g_ is observed under the vertical *F*. Fig. 7(c) and (d) show that under vertical *F*, the VBM of silicene/HGa–GaAs heterostructure is located at the K point, and the CBM is located at the Γ point, which is remarkably affected by *F*. When a negative *F* is applied, the *E*_g_ of the silicene/HGa–GaAs heterostructure disappears. Under a positive *F*, *E*_g_ begins to open at 0.3 V Å^−1^ and increases with increasing *F*.

For the freestanding silicene, an electric field *F* of 0.5 V Å^−1^ can only induce low *E*_g_ of about 20 meV,^43,44^ which limits the practical application of silicene. By contrast, the *E*_g_ values of silicene on GaAs(111) surfaces are 0.2 and 0.41 eV. The applications with external *F* cannot induce a large *E*_g_ but can realize the adjustment of *E*_g_. In addition, silicene/HGaAs heterostructures are sensitive to the negative *F*, easily lose *E*_g_, and show metallic properties.

### 3.2 Optical properties

The optical properties of silicene on semiconductors should be studied to design various optoelectronic devices on the basis of silicene applications. Having discussed the results on geometric and electronic structures of silicene on GaAs(111) surfaces, we now focus on discussing the results obtained for the optical response properties of silicene. Optical response characteristics are calculated using 33 × 33 × 1 M–P *k*-point meshes.

#### 3.2.1 Dielectric function

In optical property calculations, the real and imaginary parts of the dielectric function for the light polarized parallel and perpendicular to the silicene/HGaAs heterostructure surface are evaluated. The dielectric function is defined as the relationship between frequency and light propagation in the material. The real part of the dielectric function is closely related to the dispersion effect, and the imaginary part can reflect the absorption loss.^45,46^ The dielectric function plays a certain role in reflecting the microscopic physical process of electronic transition and optical spectral information.

**Fig. 8:**
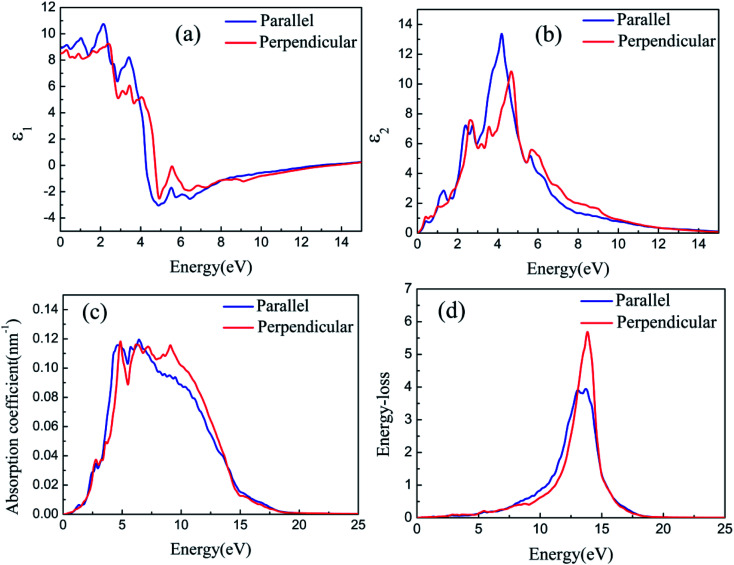
The real part (a) and imaginary part (b) of the dielectric function, the optical absorption spectra (c), and the energy loss spectra (d) with the photon energy for silicene/HAs–GaAs heterostructure.

**Fig. 9:**
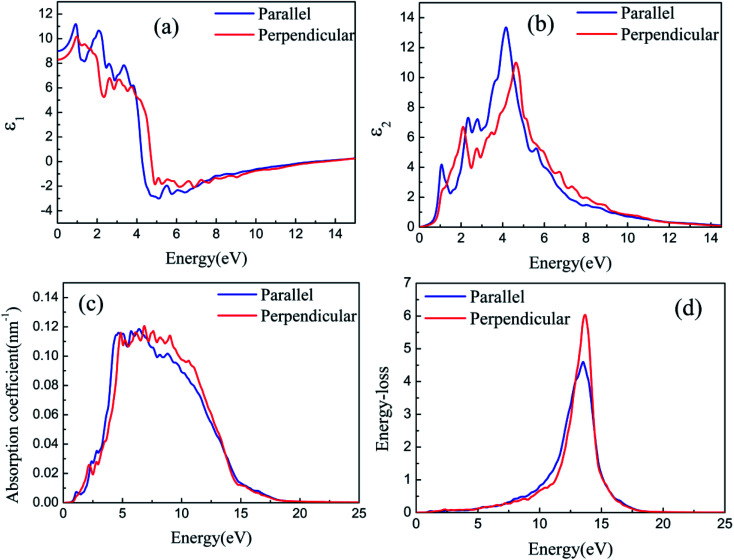
The real part (a) and imaginary part (b) of the dielectric function, the optical absorption spectra (c), and the energy loss spectra (d) with the photon energy for silicene/HGa–GaAs heterostructure.

Figs. 8(a) and 9(a) depict the real parts of the dielectric function of silicene/HAs–GaAs and silicene/HGa–GaAs heterostructures, respectively, as a function of the incident photon energy. The parallel components (*ε*_*xx*_ and *ε*_*yy*_) are represented by *ε*_∥_, and the perpendicular component (*ε*_*zz*_) is represented by *ε*_⊥_. The static values of the real part of the dielectric function are *ε*_∥_(0) = 8.92 and *ε*_⊥_(0) = 8.51 for silicene/HAs–GaAs and *ε*_∥_(0) = 8.99 and *ε*_⊥_(0) = 8.29 for silicene/HGa–GaAs. Multiple dielectric peaks are observed within 0–7 eV photon energy range. When the photon energy is over about 4 eV, the real part of the dielectric function decreases sharply due to the enhanced light absorption of the interband transition. Figs. 8(b) and 9(b) depict the imaginary parts of the dielectric function of silicene/HAs–GaAs and silicene/HGa–GaAs heterostructures, respectively, as a function of the incident photon energy. The imaginary part of the dielectric function has multiple peaks related to the interband transition in the photon energy of 0–6 eV. For the silicene/Has–GaAs heterostructure, the strongest dielectric peak appears at 4.2 and 4.7 eV for parallel and perpendicular polarizations, respectively. For the silicene/HGa–GaAs heterostructure, the strongest dielectric peak appears at 4.1 and 4.6 eV for parallel and perpendicular polarizations, respectively. Comparing the two heterostructures, the number of peaks in the imaginary part of the silicene/HAs–GaAs heterostructure is significantly more than that of the silicene/HGa–GaAs heterostructure, indicating that the silicene/HAs–GaAs heterostructure has more interband transition processes than the silicene/HGa–GaAs heterostructure.

#### 3.2.2 Absorption spectra

The calculated optical absorption spectra are shown in Figs. 8(c) and 9(c). The absorption edges of the two heterostructures are consistent with the *E*_g_ in band structures. The silicene/HAs–GaAs heterostructure is a direct *E*_g_ semiconductor. Thus, strong absorption is observed at the beginning when the photon energy is over *E*_g_, and the absorption coefficient rises steeply, reflecting direct transition processes. By contrast, for the indirect *E*_g_ semiconductor, the intrinsic absorption begins when the photon energy is over *E*_g_, and the absorption coefficient first rises to a flat region that corresponds to the indirect transition. Then, increasing photon energy results in sharply increased absorption coefficient. The strong absorption occurs, indicating the beginning of the direct transition. Notably, for the indirect *E*_g_ of the silicene/HGa–GaAs heterostructure, no evident flat region from indirect transition to direct transition is observed in the absorption spectrum because the energy of the subminimum energy valley (*K* valley) near CBM is only 0.16 eV over the minimum energy valley. Thus, the flat region representing indirect transition to direct transition is an extremely small interval.

The light response range of the heterostructure is relatively large and has multiple absorption peaks in 1–12 eV. Strong absorption peaks are distributed in the ultraviolet light region (>3.9 eV). For the silicene/Has–GaAs heterostructure, the typical absorption peaks of freestanding silicene are preserved in the absorption spectrum because the interaction between silicene and substrate is the van der Waals force. Chen *et al.* have given the absorption spectrum of the freestanding silicene layer through the first-principles calculations.^47,48^ Two typical absorption peaks are observed in the freestanding silicene layer. The first peak is a weak absorption peak caused by π–π* transition at 1.9 eV, and the second peak is the strongest absorption peak caused by σ–σ* transition at 4.4 eV. In our work, typical absorption peaks corresponding to freestanding silicene appear at 2.5 and 5 eV, respectively, which have remarkably blue-shifted compared with those of the freestanding silicene layer. It is because that the interaction between silicene and GaAs(111) substrate makes the CBM upward. The silicene/HGa–GaAs heterostructure only preserves one typical absorption peak of the freestanding silicene, and this peak is narrow and blue shifts. The maximum peak appears at about 7 eV, which corresponds to the maximum absorption peak of bulk GaAs.^49^ The above analysis shows that silicene/HGaAs heterostructures have typical optical properties of silicene and exhibit some unique optical properties (such as unique dielectric function and absorption spectrum) in the visible light irradiation range, indicating potential applications in optical transmission and optoelectronic devices.

#### 3.2.3 Electron energy loss spectra

The energy loss of electrons passing through a uniform dielectric is often described by the energy loss spectrum. The energy loss spectra of silicene/HGaAs heterostructures are given in Figs. 8(d) and 9(d). The energy loss spectra below 10 eV are caused by the interband transition around K and Γ points. The energy loss spectra in this range (below 10 eV) are relatively small, and no main loss peak is observed. The loss functions of the two heterostructures reach the maximum of around 14 eV, which are caused by the plasmon excitation. The frequency of photons for the loss function that reaches the maximum is called the plasma frequency (*ω*_p_). The energy loss spectra in the range of photon energy over *ω*_p,_ corresponds to the absorption coefficient dropping rapidly in the absorption spectra. When the incident photon energy is below 1 eV or over 20 eV, the energy loss tends to zero, showing the characteristics of transparency and corresponding to the region of the absorption coefficient reaching zero in the absorption spectrum. It can be seen from Fig. 8 and 9 that the spectra anisotropy of parallel and perpendicular directions in silicene/HGaAs heterostructures exists. The absorption peaks of the two heterostructures are concentrated in the ultraviolet region, and the energy loss peaks reflect the coupling processes between the plasmon in silicene and GaAs(111) surface.

## 4. Conclusion

In summary, we have investigated the structural, electronic, and optical properties of silicene/HGaAs heterostructures by using the first-principles method. Results show that the hydrogen intercalation successfully weakens the interaction between the silicene layer and the GaAs(111) surface and causes to open a considerable *E*_g_. The silicene/HGa–GaAs heterostructure has indirect *E*_g_, while the silicene/HAs–GaAs heterostructure has direct *E*_g_. Compared on Ga-terminated GaAs(111) surface, the silicene layer on As-terminated GaAs(111) surface has lower buckling height which is conducive to experimental growth. The electronic properties of silicene/HGaAs heterostructures can be controlled by adjusting the layer spacing *L* and the buckling height *h*, and applying biaxial strain *ε* and external electric field *F*. Adjusting *L*, *h* and applying *ε* can induce a large *E*_g_ and realize the transition between direct and indirect bandgap *E*_g_. However, the two heterostructures can only withstand a small *ε*, and a large *ε* causes the loss in *E*_g_ and transformation into metallic properties. Applying an external *F* can adjust *E*_g_ but cannot induce a large *E*_g_. In addition, silicene/HGaAs heterostructures are sensitive and easily lose *E*_g_ to become metallic under the negative perpendicular *F*. The optical properties of the two heterostructures are analyzed, and results show multiple absorption peaks in the energy range of 1–12 eV, and the typical optical properties of freestanding silicene are retained in the spectra of silicene/HGaAs heterostructures. The anisotropy of parallel and perpendicular directions in the two heterostructures exists, and the absorption and energy loss peaks are concentrated in the ultraviolet region. Our prediction results may provide guidance on the expected results of silicene/semiconductor heterostructures under experimental conditions. Theoretical observations are expected to promote the application of silicene in optoelectronic devices.

## Conflicts of interest

There are no conflicts to declare.

## Supplementary Material

## References

[cit1] Teo B. K., Sun X. H. (2007). Chem. Rev..

[cit2] Tang Q., Zhou Z. (2013). Prog. Mater. Sci..

[cit3] Rao C. N. R., Gopalakrishnan K., Maitra U. (2015). ACS Appl. Mater. Interfaces.

[cit4] Akinwande D., Petrone N., Hone J. (2014). Nat. Commun..

[cit5] Liu C., Feng W., Yao Y. (2011). Phys. Rev. Lett..

[cit6] Farokhnezhad M., Esmaeilzadeh M., Ahmadi S., Pournaghavi N. (2015). J. Appl. Phys..

[cit7] Pei Q., Zhang Y., Sha Z., Shenoy V. B. (2013). J. Appl. Phys..

[cit8] Takeda K., Shiraishi K. (1994). Phys. Rev. B: Condens. Matter Mater. Phys..

[cit9] Feng B., Ding Z., Meng S., Yao Y., He X., Cheng P., Chen L., Wu K. (2012). Nano Lett..

[cit10] Lin C., Arafune R., Kawahara K., Tsukahara N., Minamitani E., Kim Y., Takagi N., Kawai M. (2012). Appl. Phys. Express.

[cit11] Chen L., Liu C. C., Feng B., He X., Wu K. (2012). Phys. Rev. Lett..

[cit12] Chen L., Li H., Feng B., Ding Z., Qiu J., Cheng P., Wu K., Meng S. (2013). Phys. Rev. Lett..

[cit13] Qiu J., Fu H., Xu Y., Oreshkin A. I., Shao T., Hui L., Meng S., Chen L., Wu K. (2015). Phys. Rev. Lett..

[cit14] Sheng S., Wu J. B., Cong X., Li W., Gou J., Zhong Q., Cheng P., Tan P. H., Chen L., Wu K. (2017). Phys. Rev. Lett..

[cit15] Molle A., Grazianetti C., Chiappe D., Cinquanta E., Cianci E., Tallarida G., Fanciulli M. (2013). Adv. Funct. Mater..

[cit16] Cinquanta E., Scalise E., Chiappe D., Grazianetti C., van den Broek B., Houssa M., Fanciulli M., Molle A. (2013). J. Phys. Chem. C.

[cit17] Tao L., Cinquanta E., Chiappe D., Grazianetti C., Fanciulli M., Dubey M., Molle A., Akinwande D. (2015). Nat. Nanotechnol..

[cit18] Zhong H., Quhe R., Wang Y., Shi J., Lü J. (2015). Chin. Phys. B.

[cit19] Zhou S. Y., Gweon G. H., Fedorov A. V., First P. N., de Heer W. A., Lee D. H., Guinea F., Castro N. A., Lanzara A. (2007). Nat. Mater..

[cit20] Song H. J., Son M., Park C., Lim H., Levendorf M. P., Tsen A. W., Park J., Choi H. C. (2012). Nanoscale.

[cit21] Chiappe D., Scalise E., Cinquanta E., Grazianetti C., van den Broek B., Fanciulli M., Houssa M., Molle A. (2014). Adv. Mater..

[cit22] Kresse G., Furthmüller J. (1996). Phys. Rev. B: Condens. Matter Mater. Phys..

[cit23] Blanc X., Cancès É., Dupuy M. (2017). C. R. Acad. Sci. Paris, Ser. I.

[cit24] Perdew J. P., Burke K., Ernzerhof M. (1997). Phys. Rev. Lett..

[cit25] Klimeš J., Bowler D. R., Michaelides A. (2011). Phys. Rev. B: Condens. Matter Mater. Phys..

[cit26] Deak P., Aradi B., Frauenheim T., Janzen E., Gali A. (2010). Phys. Rev. B: Condens. Matter Mater. Phys..

[cit27] Luo G., Han Z., Chien C., Ko C., Wann C. H., Lin H., Shen Y., Chung C., Huang S., Cheng C., Chang C. (2010). J. Electrochem. Soc..

[cit28] Yu T., Lu Y. (2020). Phys. Chem. Chem. Phys..

[cit29] Riedl C., Coletti C., Iwasaki T., Zakharov A. A., Starke U. (2009). Phys. Rev. Lett..

[cit30] Guo Z., Oshiyama A. (2014). Phys. Rev. B: Condens. Matter Mater. Phys..

[cit31] Guo Z., Furuya S., Iwata J., Oshiyama A. (2013). Phys. Rev. B: Condens. Matter Mater. Phys..

[cit32] Prudkovskiy V. S., Iacovella F., Katin K. P., Maslov M. M., Cherkashin N. (2018). Nanotechnology.

[cit33] Zhang X., Guo Z., Cao J., Xiao X., Ding J. (2015). Acta Phys. Sin..

[cit34] Woolf D. A., Westwood D. I., Williams R. H. (1993). Appl. Phys. Lett..

[cit35] Nakatsuji H., Kamakura Y., Taniguchi K. (2003). J. Comput. Electron..

[cit36] Thompson S. E., Armstrong M., Auth C., Cea S. (2004). IEEE Electron Device Lett..

[cit37] Pereira V. M., Castro N. A. (2009). Phys. Rev. Lett..

[cit38] Levy N., Burke S. A., Meaker K. L., Panlasigui M., Zettl A., Guinea F., Castro Neto A. H., Crommie M. F. (2010). Science.

[cit39] Barraza-Lopez S., Pacheco Sanjuan A. A., Wang Z., Vanević M. (2013). Solid State Commun..

[cit40] Li X., Cao H., Guo Y., Zhou X., Yu J. (2020). Mater. Chem. Phys..

[cit41] Liu C., Feng W., Yao Y. (2011). Phys. Rev. Lett..

[cit42] Kaloni T. P., Cheng Y. C., Schwingenschlögl U. (2013). J. Appl. Phys..

[cit43] Quhe R., Fei R., Liu Q., Zheng J., Li H., Xu C., Ni Z., Wang Y., Yu D., Gao Z., Lu J. (2012). Nano Lett..

[cit44] Drummond N. D., Zólyomi V., Fal'Ko V. I. (2012). Phys. Rev. B: Condens. Matter Mater. Phys..

[cit45] Cao H., Zhou Z., Zhou X., Cao J. (2017). Comput. Mater. Sci..

[cit46] Fadaie M., Shahtahmassebi N., Roknabad M. R. (2016). Opt. Quantum Electron..

[cit47] Chen X., Jiang J., Liang Q., Meng R., Tan C., Yang Q., Sun X. (2016). J. Mater. Chem. C.

[cit48] Das R., Chowdhury S., Majumdar A., Jana D. (2014). RSC Adv..

[cit49] Yan Y., Wang Y., Ma H. (2016). Chin. J. Physiol..

